# Arthrogryposis multiplex congenita with polymicrogyria and infantile encephalopathy caused by a novel *GRIN1* variant

**DOI:** 10.1038/s41439-020-00116-8

**Published:** 2020-09-25

**Authors:** Naoto Nishimura, Tatsuro Kumaki, Hiroaki Murakami, Yumi Enomoto, Kaoru Katsumata, Katsuaki Toyoshima, Kenji Kurosawa

**Affiliations:** 1 Division of Medical Genetics, Kanagawa Children’s Medical Center, Yokohama, Japan; 2 Department of Pediatrics, National Defense Medical College, Tokorozawa, Japan; 3 Clinical Research Institute, Kanagawa Children’s Medical Center, Yokohama, Japan; 4 Department of Neonatology, Kanagawa Children’s Medical Center, Yokohama, Japan

**Keywords:** Paediatric neurological disorders, Epilepsy

## Abstract

Variants of *GRIN1*, which encodes GluN1, are associated with developmental delay, epilepsy, and cortical malformation. Here, we report a case of arthrogryposis multiplex congenita with polymicrogyria and infantile encephalopathy caused by a heterozygous variant, c.1949A>C, p.(Asn650Thr) of *GRIN1*, which could result in the disruption of the third transmembrane domain (M3) of GluN1. This case expands our understanding of the known phenotypes of *GRIN1*-related neurodevelopmental disorders.

N-methyl-D-aspartate receptors (NMDARs) are glutamate-gated ion channels that are essential for synaptic transmission and plasticity in the central nervous system (CNS). They are composed of protein tetramers, comprising two GluN1 subunits and two GluN2 subunits or a mixture of GluN2 and GluN3 subunits^1^. The GluN1 subunit is encoded by *GRIN1*, which maps to chromosome 9q34.3. Variations in *GRIN1* are associated with NMDAR functional loss or gain that leads to impaired CNS function and results in neurodevelopmental disorders, including developmental delay, epilepsy, muscle tone abnormalities, microcephaly, and cortical malformation (Table 1)^2–4^. For example, heterozygous missense *GRIN1* variants have been implicated in neurodevelopmental disorders with hyperkinetic movements and seizures (MIM 614254)^3,5^. *GRIN1* variants have also been linked to infantile encephalopathy with CNS abnormalities^6^, but they have rarely been associated with arthrogryposis multiplex congenita (AMC). Overall, little is known about the clinical phenotypes associated with *GRIN1* variant-related disruption of neuronal development, and expanding the list of known phenotypes is essential for accurate diagnosis and selection of appropriate treatment/management options.

**Table 1:** Clinical features of the proposita and other patients with *GRIN1*-related neurodevelopmental disorders.

Clinical feature	This study	Previous reports^2^ (*N* = 72)
Facial feature	None	Nonspecific
Microcephaly	+	27%
Epilepsy	+	65%
Muscular tone abnormality	+; Spasticity	Muscular hypotonia in 66% Spasticity in 40%
Movement disorder	+	48%
Polymicrogyria	+	15%
Arthrogryposis	+	Club foot and/or clenched hand in 3%

Here, we report the case of a newborn with AMC, polymicrogyria, and infantile-onset epilepsy caused by a novel *GRIN1* variant. The proband was found to have perinatal intrauterine growth restriction (IUGR) without oligohydramnios and fetal akinesia upon fetal ultrasound. She was born to healthy nonconsanguineous parents at 39 weeks of gestation, with a birth weight of 2,312 g (−1.8 standard deviation [SD]), a length of 40.4 cm (−4.0 SD), and an occipitofrontal circumference of 29.5 cm (−2.7 SD). After birth, she was transferred to our hospital due to AMC and involuntary movements. On arrival, she exhibited a narrow chest, AMC (symmetrical flexion contractures predominantly in the elbow and knee), club foot, spasticity, and respiratory distress requiring intubation. Brain magnetic resonance imaging (MRI) showed bilateral polymicrogyria in the perisylvian region (Fig. 1). After extubation, she developed epileptic seizures with episodes of desaturation. Electroencephalography revealed focal epileptic activity in the left temporal area. Treatment with diazepam, carbamazepine, and tizanidine was initiated and controlled her symptoms, including seizures and spasticity.

**Fig. 1:**
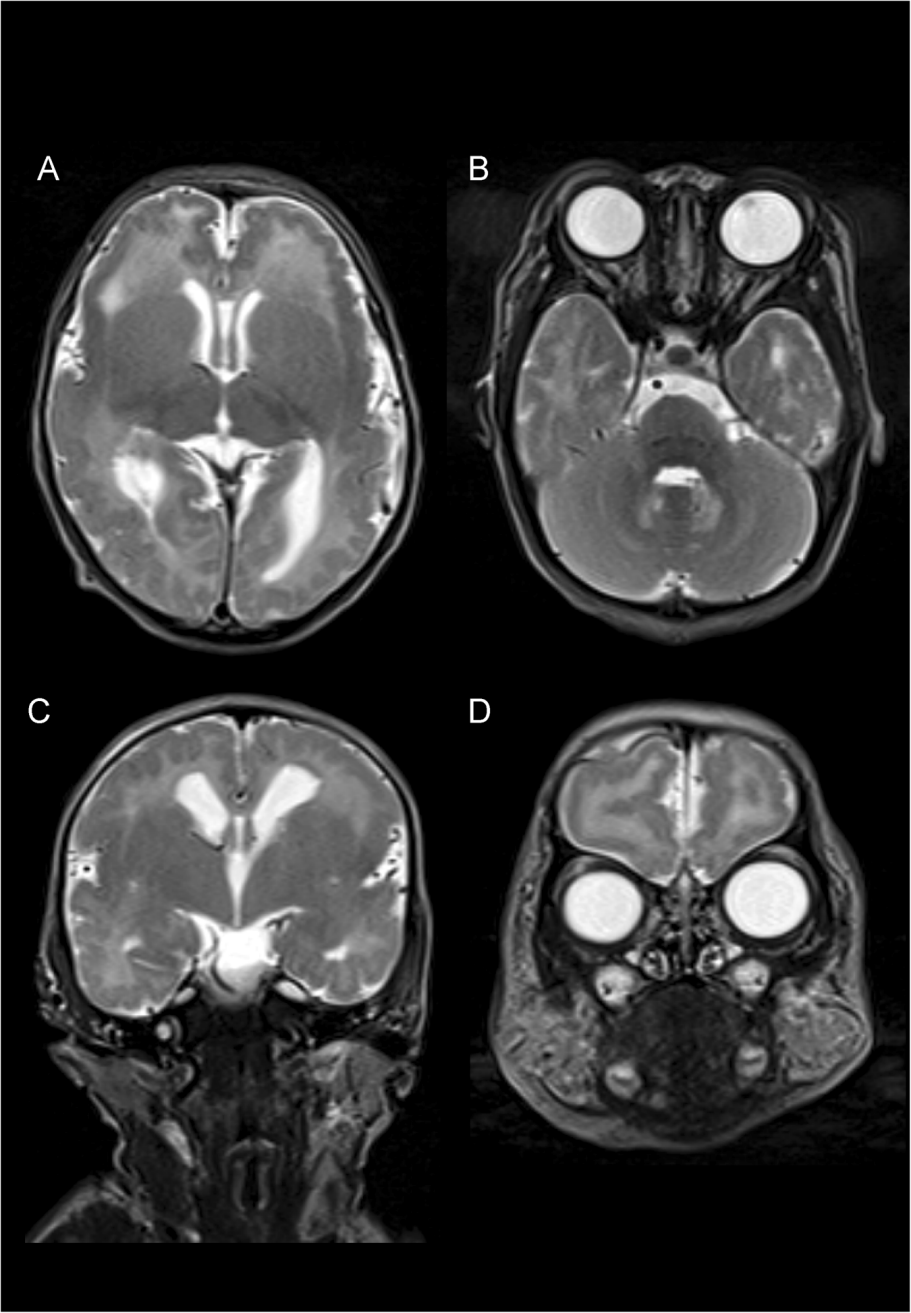
Brain MRI findings. T2-weighted axial (**a**, **b**) and coronal (**c**, **d**) brain MRI showing bilateral polymicrogyria in the perisylvian and frontal regions.

Informed consent was obtained from the parents in accordance with the Kanagawa Children’s Medical Center Review Board and Ethics Committee. Genetic analysis was performed using the TruSight One Sequencing Panel Kit on the MiSeq platform (Illumina, Inc., San Diego, CA, USA). Exome data alignment and variant calling were performed, and variant annotations were examined^7^. Targeted sequencing identified a de novo *GRIN1* heterozygous variant, NM_007327.3: c.1949A>C, p.(Asn650Thr) (Fig. 2a). The variant has not been previously reported in the general population (The Genome Aggregation Database (https://gnomad.broadinstitute.org/)). The CADD score (27.7) indicated that the variant was deleterious, and Provean (Deleterious), SIFT (Damaging), and PolyPhen-2 (Damaging) analyses predicted different types of pathogenicity. According to the American College of Medical Genetics and Genomics guidelines, the variant is pathogenic (PS2_mod + PM1 + PM2 + PM5 + PP2 + PP3)^8^.

**Fig. 2:**
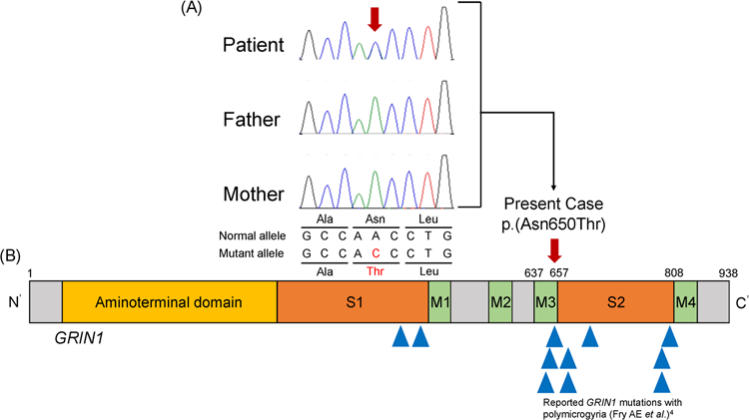
Genetic analysis and the GRIN1 domains. **a** Sanger sequencing chromatograms of a de novo heterozygous *GRIN1* variant, c.1949A>C, p.(Asn650Thr). **b** The variant found in this study is shown in the structural domains of GRIN1, including the ligand-binding site domain (S2) and the transmembrane domain (M3). The variation is located in the M3 domain. Blue triangles indicate other reported *GRIN1* variants with polymicrogyria.

The Asn650Thr variant results in a mutation in the third transmembrane domain (M3) of GluN1. The M3 domain is a highly conserved region among NMDARs and has a high impact on their functionality^9^. The M3 and the adjacent second ligand-binding (S2) domains are hot spots of *GRIN1* variations associated with polymicrogyria (Fig. 2b)^4^. In fact, the Asn650Thr variant caused a change at the same amino acid position in the M3 domain that has previously been reported in a variant causing polymicrogyria (Asn650Ile)^4^.

AMC can be caused by various conditions, mainly genetic diseases. All causes have fetal akinesia in common. The causes of fetal akinesia may be CNS, connective tissue, or skeletal abnormalities^10^. Malformations, such as arthrogryposis, club foot, and micrognathia, have been reported to occur in ~30% of patients with bilateral perisylvian polymicrogyria^11^. We hypothesize that the AMC in our patient was likely caused by a complex mechanism secondary to the neurodevelopmental deficit caused by polymicrogyria, which is an abnormality of cortical formation during early development^12^. Thus, the AMC in our patient may have been caused by a variety of different pathways, including functional disruption of NMDARs.

*GRIN1* encodes GluN1, which is expressed as a part of the NMDA receptor complex on the neuronal surface during embryonic brain development, critically influencing NMDAR function after birth^13^. Our findings indicate that alterations of *GRIN1*, especially those impacting the M3 and S2 domains, can have serious impacts on the early development and function of the CNS. Further investigation of the relationship between AMC and polymicrogyria could clarify the neurodevelopmental mechanism underlying this phenotype, especially during fetal growth.

Puduri et al. suggested that polymicrogyria with AMC may have an underlying genetic background based on a large research database of polymicrogyria^14^. They showed that typical CNS involvement and lower motor neuron defects were often observed in patients with polymicrogyria and AMC. *BICD2* and *PI4KA* are the causative genes underlying the complex phenotype, including AMC and polymicrogyria^15,16^. These genes are also responsible for other neurological features involved in peripheral nervous pathology or cerebellar hypoplasia. Our patient could move her upper and lower limbs without a decreased deep tendon reflex; however, a nerve conduction study was not performed. The brain MRI did not show cerebellar hypoplasia (Fig. 1). In line with these results, polymicrogyria with AMC is likely to be a heterogeneous disorder, and further genetic analyses are required to elucidate the complex underlying mechanisms.

In conclusion, we identified a novel *GRIN1* variant responsible for AMC and cortical abnormalities in a newborn. To our knowledge, this is the first report of severe neurodevelopmental disorders associated with AMC linked to a *GRIN1* variant. Our findings expand the known phenotypes in the spectrum of *GRIN1*-related neurodevelopmental disorders, which is essential for accurate diagnosis and development of specific therapeutic strategies.

## Data Availability

The relevant data from this Data Report are hosted at the Human Genome Variation Database at 10.6084/m9.figshare.hgv.2900.

## References

[1] Paoletti, P., Bellone, C. & Zhou, Q. NMDA receptor subunit diversity: impact on receptor properties, synaptic plasticity and disease. *Nat. Rev. Neurosci.* **14**, 383–400 (2013). 10.1038/nrn3504 23686171

[2] Platzer, K. & Lemke, J. R. *GRIN1*-*Related Neurodevelopmental Disorder*. (eds Adam, M. P., Ardinger, H. H., Pagon, R. A., Wallace, S. E., Bean, L. J. H., Stephens, K. et al.) (GeneReviews((R)), Seattle, 2019). 31219694

[3] Lemke JR (2016). Delineating the *GRIN1* phenotypic spectrum: a distinct genetic NMDA receptor encephalopathy. Neurology.

[4] Fry AE (2018). De novo mutations in *GRIN1* cause extensive bilateral polymicrogyria. Brain.

[5] Hamdan FF (2011). Excess of *de novo* deleterious mutations in genes associated with glutamatergic systems in nonsyndromic intellectual disability. Am. J. Hum. Genet..

[6] Ohba C (2015). *GRIN1* mutations cause encephalopathy with infantile-onset epilepsy, and hyperkinetic and stereotyped movement disorders. Epilepsia.

[7] Murakami H, Enomoto Y, Tsurusaki Y, Sugio Y, Kurosawa K (2020). A female patient with X-linked Ohdo syndrome of the Maat-Kievit-Brunner phenotype caused by a novel variant of MED12. Congenit. Anom..

[8] Richards S (2015). Standards and guidelines for the interpretation of sequence variants: a joint consensus recommendation of the American College of Medical Genetics and Genomics and the Association for Molecular Pathology. Genet Med..

[9] Leventer RJ (1999). Clinical and imaging features of cortical malformations in childhood. Neurology.

[10] Hall JG (2014). Arthrogryposis (multiple congenital contractures): diagnostic approach to etiology, classification, genetics, and general principles. Eur. J. Med. Genet..

[11] Jansen A, Andermann E (2005). Genetics of the polymicrogyria syndromes. J. Med. Genet..

[12] Yuan H, Erreger K, Dravid SM, Traynelis SF (2005). Conserved structural and functional control of N-methyl-D-aspartate receptor gating by transmembrane domain M3. J. Biol. Chem..

[13] Chen W (2017). Grin1 mutation associated with intellectual disability alters NMDA receptor trafficking and function. J. Hum. Genet..

[14] Poduri A (2010). The syndrome of perisylvian polymicrogyria with congenital arthrogryposis. Brain Dev..

[15] Peeters K (2013). Molecular defects in the motor adaptor BICD2 cause proximal spinal muscular atrophy with autosomal-dominant inheritance. Am. J. Hum. Genet..

[16] Pagnamenta AT (2015). Germline recessive mutations in *PI4KA* are associated with perisylvian polymicrogyria, cerebellar hypoplasia and arthrogryposis. Hum. Mol. Genet..

